# Development of Injectable Polydactyly-Derived Chondrocyte Sheets

**DOI:** 10.3390/ijms22063198

**Published:** 2021-03-21

**Authors:** Shiho Wasai, Eriko Toyoda, Takumi Takahashi, Miki Maehara, Eri Okada, Ryoka Uchiyama, Tadashi Akamatsu, Masahiko Watanabe, Masato Sato

**Affiliations:** 1 Department of Orthopaedic Surgery, Surgical Science, Tokai University School of Medicine, 143 Shimokasuya, Isehara, Kanagawa 259-1193, Japan; sw12345@tsc.u-tokai.ac.jp (S.W.); etoyoda@tokai-u.jp (E.T.); takumi.takahashi22@gmail.com (T.T.); m-maehara@tsc.u-tokai.ac.jp (M.M.); okada-eri@tokai-u.jp (E.O.); 9bmrm002@mail.u-tokai.ac.jp (R.U.); masahiko@is.icc.u-tokai.ac.jp (M.W.); 2 Center for Musculoskeletal Innovative Research and Advancement (C-MiRA), Graduate School of Medicine, Tokai University, 143 Shimokasuya, Isehara, Kanagawa 259-1193, Japan; 3 Department of Plastic Surgery, Surgical Science, Tokai University School of Medicine, 143 Shimokasuya, Isehara, Kanagawa 259-1193, Japan; akamatu@is.icc.u-tokai.ac.jp

**Keywords:** cartilage regeneration, cell sheet, osteoarthritis, minimally invasive treatment

## Abstract

We are conducting a clinical study of the use of allogeneic polydactyly-derived chondrocyte sheets (PD sheets) for the repair of articular cartilage damage caused by osteoarthritis. However, the transplantation of PD sheets requires highly invasive surgery. To establish a less invasive treatment, we are currently developing injectable fragments of PD sheets (PD sheets-mini). Polydactyly-derived chondrocytes were seeded in RepCell™ or conventional temperature-responsive inserts and cultured. Cell counts and viability, histology, enzyme-linked immunosorbent assay (ELISA), quantitative real-time polymerase chain reaction (qPCR), and flow cytometry were used to characterize PD sheets-mini and PD sheets collected from each culture. To examine the effects of injection on cell viability, PD sheets-mini were tested in four experimental conditions: non-injection control, 18 gauge (G) needle, 23G needle, and syringe only. PD sheets-mini produced similar amounts of humoral factors as PD sheets. No histological differences were observed between PD sheets and PD sheets-mini. Except for *COL2A1*, expression of cartilage-related genes did not differ between the two types of PD sheet. No significant differences were observed between injection conditions. PD sheets-mini have characteristics that resemble PD sheets. The cell viability of PD sheets-mini was not significantly affected by needle gauge size. Intra-articular injection may be a feasible, less invasive method to transplant PD sheets-mini.

## 1. Introduction

Articular cartilage is a unique tissue that comprises mainly extracellular matrix (ECM), in the form of collagens and proteoglycans, and has a hypocellular and avascular structure [1]. Given its tissue properties, articular cartilage can tolerate intensive and repetitive physical stress but, once damaged, is difficult to repair [2]. Diffuse damage to articular cartilage gradually promotes joint degeneration, which eventually leads to osteoarthritis (OA), especially in the knee joint.

OA of the knee (OAK) is the most common joint disease, involves cartilage defects of varied depth and breadth, and is the most frequent cause of knee pain and dysfunction. OA progresses slowly with age, reduces the quality of life and work productivity, and ultimately imposes socioeconomic costs worldwide [3]. The Framingham osteoarthritis study found that the age-standardized prevalence of OAK reached 19.2% (18.6% for men and 19.3% for women) in people older than 45 years. In Japan, where the aging population is increasing, the number of OAK patients is expected to continue increasing in the future [4,5,6].

Current methods of treatment for OAK include patient education, exercise, oral treatments, conservative therapy by intra-articular injection of hyaluronic acid or steroids, and surgical treatments, including arthroscopic debridement of the knee joint, osteotomy around the knee such as a high tibial osteotomy, unicompartmental knee arthroplasty, and total knee arthroplasty [7,8].

Autologous chondrocyte implantation (ACI) is performed as a regenerative treatment for cartilage defects. However, most of the indications for ACI are cartilage defects caused by trauma and osteochondritis dissecans, and ACI does not have a sufficient therapeutic effect on OAK. Moreover, because OAK is characterized by heterogeneous cartilage defects, no fully effective regenerative treatment has been developed to stop the progression of OAK or to induce cartilage regeneration [9,10,11].

Cell sheet engineering is a technique that enables the collection of cells in sheet form simply by lowering the temperature from 37 °C to below 32 °C without using enzymes such as trypsin and growing the cells in a temperature-responsive culture dish with the temperature-responsive polymer poly (N-isopropyl acrylamide) [12]. Cell sheets have been applied as a regenerative treatment of various tissues including the cornea [13], esophagus [14], myocardium [15], and periodontium [16]. We have been engaged in research on cartilage regenerative medicine using this cell sheet engineering since 2004. At present, autologous chondrocyte sheet transplantation is available as an advanced medical care B for the treatment of OAK in Japan. However, two surgical procedures are required to fabricate and transplant an autologous chondrocyte sheet. In addition, the proliferative capacity of adult chondrocytes varies greatly among individuals. To overcome these issues, we explored the possibility of using allogeneic cell sources [17].

By focusing on the immunotolerant properties of cartilage that has already been reported [18], particulated juvenile cartilage implants (De Novo ^®^ NT Graft; Zimmer, Warsaw, IN, USA) are used clinically in the United States [19]. However, the use of this product in Japan is difficult, due to the difficulty in ensuring traceability. Therefore, we focused on surgical remains obtained from patients with polydactyly as a source of allogeneic chondrocytes.

We are currently conducting a clinical study of the use of allogeneic polydactyly-derived chondrocyte sheets (PD sheets) for articular cartilage repair [20]. However, the transplantation of PD sheets requires open-knee surgery and is currently performed in conjunction with open-wedge high tibial osteotomy, which is highly invasive.

**Figure 1:**
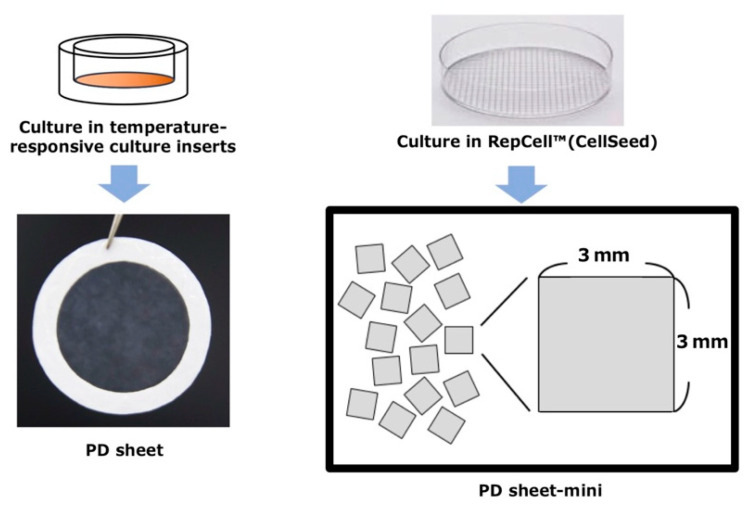
Fabrication of polydactyly-derived chondrocyte (PD) sheets and PD sheets-mini. PD sheets and PD sheets-mini were fabricated in temperature-responsive culture inserts called UpCell® and temperature-responsive culture dishes called RepCell™ (CellSeed Inc.), respectively. RepCell™ is a unique culture dish with grid walls etched onto the culture surface creating 3 mm × 3 mm grids.

Injection is a promising approach to deliver substances for less-invasive types of regenerative medicine. To establish a less-invasive treatment for OAK, we are currently developing injectable PD sheets fabricated in RepCell™ plates (CellSeed Inc., Tokyo, Japan), a temperature-responsive culture dish with 3 mm × 3 mm grid walls etched into the culture surface. Our aim is to develop sheet transplantation treatments that can be delivered through needle injection into the knee joint (Figure 1).

The purposes of this study were to examine the potential clinical applications of injectable fragments of PD sheets (PD sheets-mini) by comparing their sheet characteristics with those of conventional PD sheets and to examine the effects of PD sheets-mini injected using needles of different gauge size on cell viability.

## 2. Results

### 2.1. Comparison of Sheet Characteristics of Polydactyly-Derived Chondrocyte (PD) Sheets and PD Sheets-Mini

#### 2.1.1. Cell Count and Viability

**Figure 2:**
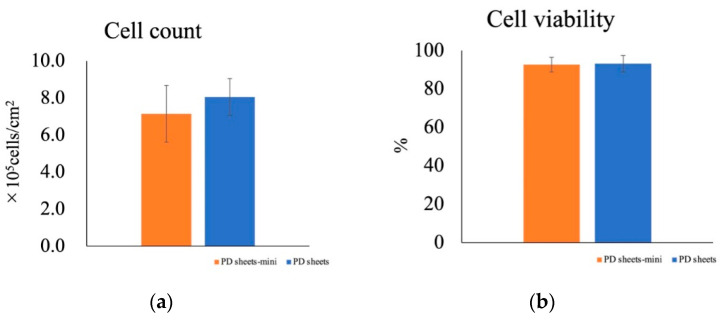
Comparison of cell count and cell viability for PD sheets and PD sheets-mini. Values are expressed as mean ± standard deviation. (**a**) Cell count of PD sheets and PD sheets-mini. (**b**) Cell viability of PD sheets and PD sheets-mini. The cell counts and cell viability did not differ between the two types of cell sheets.

After culturing for 2 weeks, both PD sheets-mini and PD sheets were collected, and the cell count corrected by the area of the culture surface was calculated. The cell count and viability did not differ significantly between PD sheets-mini and PD sheets (Figure 2a,b).

#### 2.1.2. Flow Cytometric Analysis

**Figure 3:**
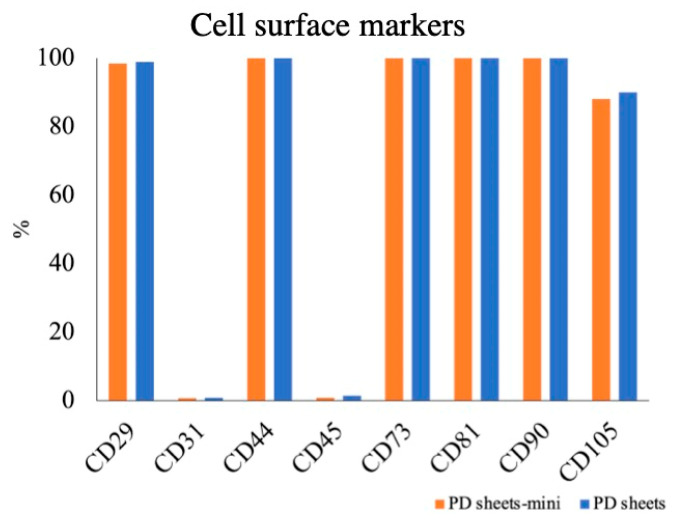
Flow cytometric analysis comparing PD sheets and PD sheets-mini. Both sheets were positive for mesenchymal stem cell markers such as CD29, CD44, CD73, CD81, CD90, and CD105, and negative for vascular endothelium and blood cell markers such as CD31 and CD45.

PD sheets-mini and PD sheets showed similar surface markers (Figure 3). Both sheets were negative for CD31, a vascular endothelium marker, and CD45, a blood cell marker: PD sheets-mini CD31 0.7%; CD45, 0.8%; PD sheets CD31, 0.8%; CD45, 1.4%. Both types of sheet were positive for the mesenchymal stem cell (MSC) markers CD29, CD44, CD73, CD81, CD90, and CD105: PD sheets-mini CD29, 98.4%; CD44, 99.9%; CD73, 99.9%; CD81, 100%; CD90, 99.9%; CD105, 88.1%; PD sheets CD29, 98.9%; CD44, 99.9%; CD73, 100%; CD81, 100%; CD90, 99.9%; CD105, 90.0%.

#### 2.1.3. Measurement of the Amounts of Humoral Factors

**Figure 4:**
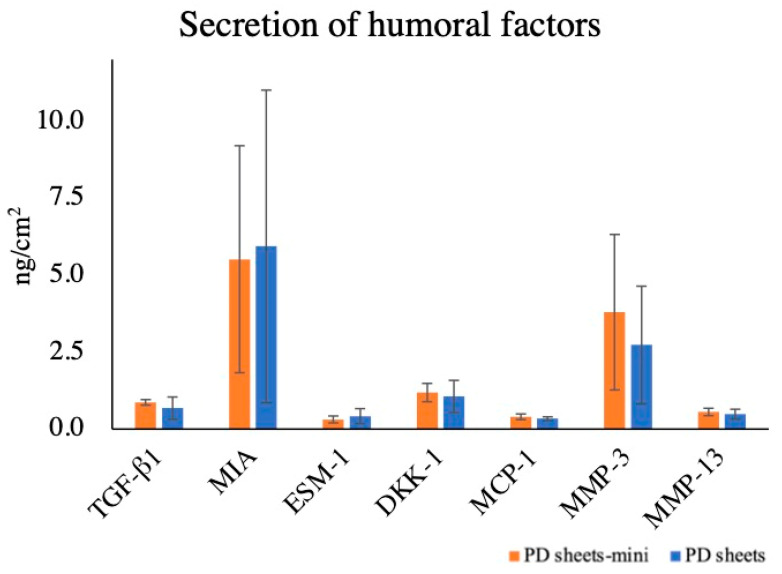
Amounts of secreted humoral factors. The amounts of transforming growth factor beta-1 (TGF-β1), melanoma inhibitory activity (MIA), endothelial cell-specific marker 1 (ESM-1), Dickkopf-1 protein (DKK-1), monocyte chemoattractant protein 1 (MCP-1), matrix metalloproteinase (MMP)-3, and MMP-13, humoral factors related to cartilage, did not differ significantly between PD sheets-mini and PD sheets.

The amounts of humoral factors were corrected by the culture surface area (Figure 4). There was no significant difference between PD sheets-mini and PD sheets in the amounts of transforming growth factor beta-1 (TGF-β1): PD sheets-mini 0.87 ± 0.09 ng/cm^2^ and PD sheets 0.68 ± 0.36 ng/cm^2^. PD sheets-mini produced similar amounts of melanoma inhibitory activity (MIA) as PD sheets: PD sheets-mini 5.52 ± 3.69 ng/cm^2^ and PD sheets 5.93 ± 5.07 ng/cm^2^. Similarly, the amounts of humoral factors produced did not differ between PD sheets and PD sheets-mini for the following markers: endothelial cell-specific marker 1 (ESM-1), PD sheets-mini, 0.31 ± 0.11 ng/cm^2^ and PD sheets, 0.42 ± 0.24 ng/cm^2^; Dickkopf-1 protein (DKK-1) PD sheets-mini, 1.19 ± 0.30 ng/cm^2^ and PD sheets 1.06 ± 0.52 ng/cm^2^; and monocyte chemoattractant protein 1 (MCP-1), PD sheets-mini, 0.40 ± 0.09 ng/cm^2^ and PD sheets 0.34 ± 0.06 ng/cm^2^. The amounts of enzymes that decompose the extracellular matrix, matrix metalloproteinase (MMP)-3 and MMP-13, also did not differ between PD sheets and PD sheets-mini. The respective values were PD sheets-mini, 3.79 ± 2.53 ng/cm^2^ and PD sheets 2.73 ± 1.91 ng/cm^2^ and PD sheets-mini, 0.56 ± 0.12 ng/cm^2^ and PD sheets 0.48 ± 0.16 ng/cm^2^.

#### 2.1.4. Gene Expression Analysis

**Figure 5:**
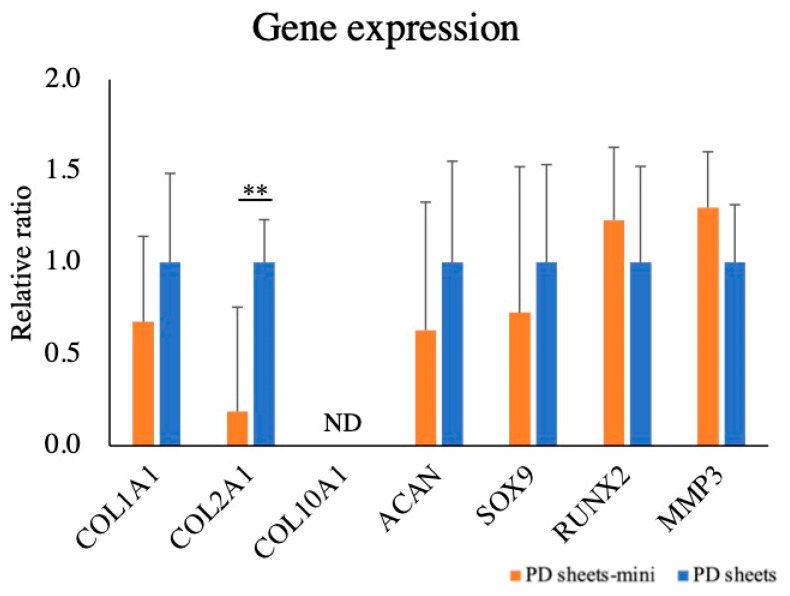
Cartilage-related gene expression analysis in PD sheets and PD sheets-mini. The relative gene expression of PD sheets was set at 1.0. The values are expressed as mean ± standard deviation. ** *p* < 0.01; ND, not detected. Only the expression of *COL2A1* differed between PD sheets and PD sheets-mini.

The relative gene expression of PD sheets was set at 1.0. The relative ratios of gene expression of PD sheets-mini were 0.68 times for *COL1A1*, 0.19 times for *COL2A1*, 0.73 times for *SOX9*, 0.63 times for *ACAN*, 1.23 times for *RUNX2*, and 1.30 times for *MMP3*. Except for *COL2A1*, the expression of cartilage-related genes did not differ between PD sheets and PD sheets-mini. The expression of *COL10A1* was not detected (Figure 5).

#### 2.1.5. Histological Analysis

**Figure 6:**
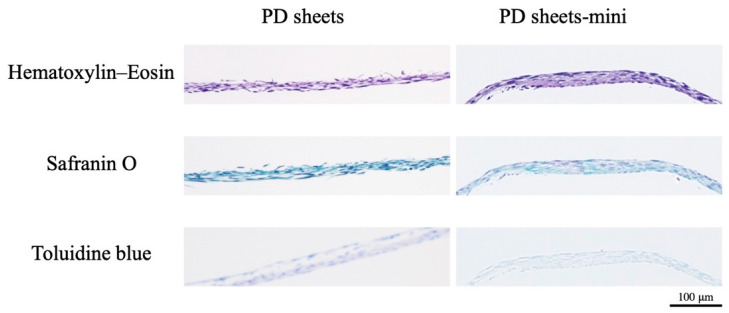
Histological analysis of PD sheets and PD sheets-mini. Sections of both PD sheets and PD sheets-mini were stained with hematoxylin–eosin, safranin O, and toluidine blue (×10, scale bar = 100 μm).

Sections of both PD sheets and PD sheets-mini showed similar multiple stratification of chondrocytes derived from polydactyly tissues. Both PD sheets-mini and PD sheets were stained weakly or not at all with Safranin O and toluidine blue. Histological analysis revealed no differences between PD sheets and PD sheets-mini (Figure 6).

### 2.2. Effects of Injection of PD Sheets-Mini on Cell Viability

**Figure 7:**
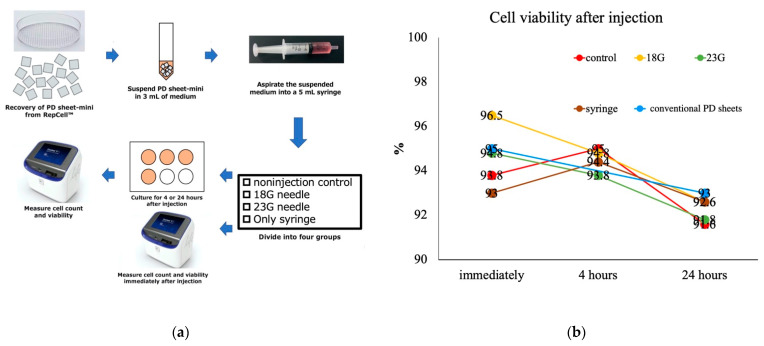
Evaluation of the effect of injection on cell viability of PD sheets-mini. (**a**) To examine the effect of injection on cell viability, the PD sheets-mini suspension was examined under four conditions: non-injection control, 18 gauge (G) needle injection, 23G needle injection, and syringe injection, and cell viability was measured at 0, 4, and 24 h after injection. At 0 and 4 h after injection, cell viability of conventional PD sheets was compared. (**b**) Comparison of cell viability of PD sheets-mini between the four conditions immediately after, 4 h, and 24 h after injection. Cell viability decreased with time but did not differ significantly between the four conditions at any time.

Cell viability decreased with time after injection but did not differ significantly between the four injection conditions immediately after (*p* = 0.748), 4 h (*p* = 0.987), and 24 h (*p* = 0.994) after injection. Cell viability of the conventional PD sheets after detachment did not differ between the four conditions (Figure 7a,b).

## 3. Discussion

Cartilage regeneration is difficult when induced only with concentrated humoral factors without cells, and the presence of cells producing cartilage-related humoral factors is considered to be important. Cell transplantation therapy by injection is a promising approach for less-invasive regenerative medicine, but the retention of transplanted cells at the injected sites is an important issue [21].

In cell sheet engineering, cells can be collected in sheets without the need for enzymes such as trypsin. Cell sheets of most types of cells can be transplanted while retaining the extracellular matrix and cell adhesion factors.

Wang et al. reported that the ability for cell attachment and proliferation was maintained even after MSC sheets were fragmented and injected through a needle, and this method may be superior to that using dissociated MSCs [21]. We believe that transplanted cells engraft at the cartilage defect sites or in the vicinity, even temporarily, and that humoral factors secreted from the cells are important for cartilage regeneration. The results of gene expression and histological evaluation suggest that dedifferentiation of chondrocytes occurs in both PD sheet and PD sheet-mini. We consider that cartilage repair is promoted by the recipient’s chondrocytes, which are influenced by the humoral factors secreted by both sheets, rather than replacing both of the transplanted sheets with cartilage tissue.

In conducting clinical studies on the transplantation of autologous chondrocyte sheets [20] and polydactyly-derived chondrocyte sheets for articular cartilage regeneration, we have reported the characteristics and therapeutic effects of both types of sheet in animal experiments using mini-pigs, rabbits, and rats [22,23,24,25]. Our previous flow cytometric analysis studies have shown that PD sheets express MSC markers and our histological analyses have shown that PD sheets are multiply stratified without stacking, as in autologous chondrocyte sheets [17]. Similar results to these studies were obtained for both PD sheets and PD sheets-mini in this study.

MIA and TGF-β1 secreted from autologous chondrocyte sheets and PD sheets play an important role in cartilage differentiation and repair. MIA, which is a cartilage differentiation marker and anabolic factor, modulates chondrogenic differentiation via MIA-regulated extracellular signal-regulated kinase (ERK)-signaling [26]. TGF-b is known to be very beneficial for cartilage as it stimulates chondrocytes to induce elevation of proteoglycan and collagen type II production and also counteracts the main catabolic factors in OA [27]. Importantly, more TGF-β1 is secreted from PD sheets than from autologous chondrocyte sheets [17,28]. We also measured the production of ESM-1 and DKK-1, which we previously reported as humoral factors whose production correlates positively with the effectiveness of PD sheets for articular cartilage repair. ESM-1 is a proteoglycan involved in angiogenesis and is expressed in the vascular endothelium and adipocytes. DKK-1 is a protein that has an inhibitory effect on the Wnt signaling pathway and is involved in bone metabolism and differentiation [29]. In this study, the secretion of these humoral factors did not differ significantly between PD sheets and PD sheets-mini and, therefore, we consider that both sheets would have an equivalent effect of cartilage regeneration.

MCP-1, a chemokine with monocyte/macrophage migration activity, is also called C–C chemokine ligand 2 (CCL2). CCL2 provokes an inflammatory response through the C–C chemokine receptor 2 (CCR2), and the CCL2–CCR2 signal plays a role in cartilage degeneration. However, Jablonski et al. reported that, in the absence of CCR2, CCL2 may be required for cartilage regeneration [30]. Based on the report by Jablonski et al., we measured MCP-1 production in this study, but found no significant difference in MCP-1 production between PD sheet and PD sheet-mini.

MMP-3 and MMP-13 are known as catabolic factors that lead to cartilage degeneration by degrading ECM [31,32]. We have reported that PD sheets produce less MMP-3 than adult autologous chondrocytes, suggesting that polydactyly-derived chondrocytes have an advantage as a cell source [17]. In this study, there was no significant difference in the production of MMP-3 and MMP-13 by PD sheets-mini compared with the conventional PD sheets.

For all humoral factors measured in this study, we found no significant differences in the amounts secreted between both types of cell sheet. The new injection form of the chondrocyte sheets used in this study (PD sheets-mini) had similar characteristics as the PD sheets. However, the expression of *COL2A1* was lower in the PD sheets-mini, and this may reflect the culture of sheets in a flat culture dish instead of an insert, unlike in conventional PD sheets. From the results of clinical studies and accompanying animal experiments that we have conducted so far, we consider that the humoral factors secreted by the transplanted chondrocyte sheets play a more important role in the regeneration of articular cartilage than the genes expressed by the sheets [20,28,29].

Injection of MSCs has been reported to have no effects on the trilineage differentiation of osteogenic, adipogenic and chondrogenic cell lines, or on cell viability [33], although it has been suggested that apoptosis may occur early after injection through a 21 gauge (G) or 23G needle [34]. However, those studies examined this issue at the cellular level, and no reports have examined the effects of injection of mini-tissues such as cell sheets.

Our findings that the injection condition had almost no effect on the cell viability of PD sheets-mini suggest that injection of PD sheets-mini is feasible. Even if cell apoptosis occurs after injection through a 21G or 23G needle, the size of the needle usually used for arthrocentesis of knee is 18G, which should have less effect on cells.

PD sheets-mini have sheet characteristics that closely resemble those of PD sheets. Our results suggest that the cell viability of PD sheets-mini is not significantly affected by the clinically relevant needle gauge size. Intra-articular injection may be a feasible method for the administration of PD sheets-mini as a less-invasive method. In the future, it is necessary to examine the effect of cartilage treatment of PD sheets-mini in vivo.

## 4. Materials and Methods

### 4.1. Collection of Polydactyly-Derived Chondrocytes

Cartilage tissue was obtained from six polydactyly patients (average age, 15.5 months; range, 12–23 months; three boys and three girls) who underwent surgery at Tokai University Hospital.

Cartilage tissue was finely minced with scissors and treated with the following procedure to isolate chondrocytes from cartilage tissue. The minced cartilage tissue was incubated in Dulbecco’s modified Eagle’s medium/F12 (DMEM/F12; Gibco, Grand Island, NY, USA) supplemented with 20% fetal bovine serum (FBS; SAFC Biosciences, Lenexa, KS, USA), 1% antibiotic–antimycotic solution (AB; Gibco), and 5 mg/mL collagenase type 1 (CLS1; Worthington, Mannheim, Germany) for 1.5 h at 37 °C in a humidified atmosphere of 5% CO_2_ and 95% air. The collected cell suspension was washed and passed through a 100 μm strainer (BD Falcon, Franklin Lakes, NJ, USA).

The isolated chondrocytes were seeded at a density of 1 × 10^4^ cells/cm^2^ in six-well culture plates (Corning, Corning, NY, USA) in DMEM/F12 supplemented with 20% FBS and 1% AB, and incubated at 37 °C. After 4 days, 100 μg/mL ascorbic acid (Nissin Pharmaceutical, Yamagata, Japan) was added to the medium, and the medium was replaced every 3 or 4 days thereafter. When the chondrocytes reached confluency, they were passaged once or twice, and passaged chondrocytes were cryopreserved at −180 °C.

### 4.2. Fabrication of PD Sheets and PD Sheets-Mini

PD sheets and PD sheets-mini were fabricated using a temperature-responsive culture insert and RepCell™ plate (CellSeed Inc.). The latter is a unique temperature-responsive culture plate with 3 mm × 3 mm grid walls etched into the culture surface. The areas of the culture dishes were 4.2 cm^2^ and 8.8 cm^2^, respectively (Figure 1).

To fabricate PD sheets, cryopreserved chondrocytes isolated from polydactyly patients were thawed, passaged once, and then seeded in temperature-responsive culture inserts (CellSeed Inc.) at 1 × 10^4^ cells/cm^2^. After culturing for 2 weeks with the medium changed every 3 or 4 days, the culture plates were allowed to stand at 25 °C for 30 min to detach PD sheets from the inserts, and each sheet was collected by hooking it onto a paper ring. PD sheets were checked visually to confirm the strength and to identify any tearing.

PD sheets-mini were fabricated using a method similar to that for PD sheets. The lysed chondrocytes were passaged and then seeded at 1 × 10^4^ cells/cm^2^ in RepCell™ plates. After culturing for 2 weeks with the medium changed every other day, each RepCell™ plate was kept at 25 °C for 30 min, and multiple 3 mm × 3 mm-sized fragments of PD sheets were collected (Figure 1).

### 4.3. Comparison of Sheet Characteristics between PD Sheets and PD Sheets-Mini

#### 4.3.1. Cell Count and Viability

PD sheets and PD sheets-mini were washed in Dulbecco’s phosphate-buffered saline (DPBS; Gibco). Each type of sheet was then incubated in TripLE Express^®^ (Gibco) at 37 °C for 15 min and centrifuged at 1500 rpm for 5 min. The sheets were resuspended in 0.25 mg/mL Collagenase P (Roche, Basel, Switzerland) at 37 °C for up to 30 min and then centrifuged at 1500 rpm for 5 min. Cells isolated from both types of sheets were resuspended in DMEM/F12, and cell count and viability were determined using trypan blue exclusion.

#### 4.3.2. Flow Cytometric Analysis

After the cell count and viability were measured, isolated cells were washed with DPBS containing 0.2% bovine serum albumin (BSA; Sigma-Aldrich, St. Louis, MO, USA) and 1 mM ethylenediaminetetraacetic acid (EDTA; Gibco). About 1.5 × 10^5^ cells were mixed in each tube with the following antibodies: hCD31–fluorescein isothiocyanate (FITC) (clone: 5.6E; Beckman Coulter, Brea, CA, USA), hCD44–FITC (clone: G44-26; BD Biosciences, Franklin, NJ, USA), hCD45–FITC (clone: J33; Beckman Coulter), hCD73–FITC (clone: AD2; BD Biosciences), hCD81–FITC (clone: JS-81; BD Biosciences), hCD90–allophycocyanin (clone: 5E10; BD Biosciences), and CD105–phycoerythrin (clone: 266; BD Biosciences).

The cells were incubated for 90 min at 4 °C and then washed with DPBS containing 0.2% BSA and 1 mM EDTA. Fluoroprobe-labeled mouse IgG1 antibody (Beckman Coulter) and rat IgG2b antibody (BioLegend, San Diego, CA, USA) were used as negative controls. Stained cells were analyzed using a FACS Verse TM cell sorter (BD Biosciences).

#### 4.3.3. Measurement of Humoral Factors

PD sheets and PD sheets-mini fabricated as described above were used. PD sheets collected by hooking on paper rings were cultured for 24 h in 3 mL of DMEM/F12 supplemented with 5% FBS and 1% AB in an adherent culture dish. PD sheets-mini were harvested from RepCell™ plates after the plate was left at room temperature for more than 30 min. PD sheets-mini were suspended in 3 mL of DMEM/F12 supplemented with 5% FBS and 1% AB, and cultured for 24 h in a culture dish. Each supernatant was collected and centrifuged at 15,000× *g* for 10 min to remove cell debris. The concentrations of TGF-β1, MIA, ESM-1, DKK-1, MCP-1, MMP-3, and MMP-13 were measured using the enzyme-linked immunosorbent assays (ELISAs) described below.

TGF-β1, MIA, and ESM-1 concentrations were measured using their respective single-sample ELISA kits: Human TGF-β1 Quantikine ELISA Kit (R&D Systems, Minneapolis, MN, USA), MIA ELISA (Roche), and Human ESM-1 ELISA Kit (Abcam, Cambridge, UK). The other humoral factors were measured using Quantibody Multiplex ELISA Array (RayBiotech Life, Inc., Norcross, GA, USA).

The signal detected for blank medium containing 5% FBS was subtracted to adjust for proteins contained in FBS. Measurements were repeated at least twice for each donor, and averages were used. The amount of humoral factor per area of the culture surface was calculated.

#### 4.3.4. Gene Expression Analysis

Each of the PD sheets and PD sheets-mini fabricated by culturing for 2 weeks was disrupted in TRIzol Reagent (Life Technologies, Carlsbad, CA, USA) and crushed at 1500 rpm for 3 min using a SHAKE Master Neo instrument (Bio Medical Science, Tokyo, Japan). Total RNA was extracted using an RNeasy Mini Kit (Qiagen, Hilden, Germany). Next, first-strand cDNA was reverse transcribed from 1 mg of total RNA and treated with a QuantiTect Reverse Transcription Kit (Qiagen) in a SimpliAmp Thermal Cycler (Thermo Fisher Scientific, Waltham, MA, USA) at 42 °C for 15 min and 95 °C for 3 min. Quantitative real-time polymerase chain reaction (qPCR) was performed using an Applied Biosystems 7300 Real-Time PCR system (Applied Biosystems, Waltham, MA, USA). The SYBR™ Green primers used are shown in Table 1. Glyceraldehyde-3-phosphate dehydrogenase (GAPDH) primers were used to normalize the samples.

#### 4.3.5. Histological Analysis

The harvested PD sheets and PD sheets-mini were fixed with 20% formalin (Wako Pure Chemical, Osaka, Japan) and embedded in paraffin wax. The embedded tissue was cut into 20-μm-thick sections, which were deparaffinized and stained with hematoxylin–eosin, Safranin O, and toluidine blue according to standard methods.

### 4.4. Analysis of the Effect of Injection of PD Sheets-Mini on Cell Viability

PD sheets-mini harvested from one RepCell™ plate were suspended in 3 mL of medium. To analyze the effect of injection on cell viability, suspensions of PD sheets-mini were tested for the following four injection conditions: non-injection control, 18G needle, 23G needle, and syringe only. For the injection condition, a 3 mL suspension was aspirated slowly into a 5 mL syringe and then injected through a needle or the syringe itself.

Cell viability was examined using the trypan blue exclusion assay at 0, 4, and 24 h after injection. Cell viability was compared among the four injection conditions, and also between PD sheets-mini and after detachment of cells from PD sheets at 0 and 24 h (Figure 7a).

### 4.5. Statistical Analysis

Significant differences between groups were identified using the Mann–Whitney *U* test for non-normally distributed quantitative data. The Kruskal–Wallis test was used to detect significant differences between three groups or more. All statistical analyses were performed using IBM SPSS Statistics (v. 25.0; IBM, Armonk, NY, USA). Probability values of <0.05 were considered to be significant.

## 5. Conclusions

PD sheets-mini have sheet characteristics that closely resemble those of PD sheets. Our results suggest that the cell viability of PD sheets-mini is not significantly affected by injection using a clinically relevant needle gauge size. Intra-articular injection may be a feasible way to administer PD sheets-mini in a less-invasive method as a point-of-care treatment for OAK.

## 6. Patents

The work reported in this study is patent pending; the application number is 2020-148200 in Japan, and the application date was 3 September 2020.

## Figures and Tables

**Table 1:** Genes and their primers used for quantitative real-time polymerase chain reaction (qPCR).

Genes	Forward Primer Sequence	Reverse Primer Sequence
*ACAN*	AGGAGACAGAGGGACACGTC	TCCACTGGTAGTCTTGGGCAT
*COL1A1*	GTCGAGGGCCAAGACGAAG	CAGATCACGTCATCGCACAAC
*COL2A1*	GTGGAGCAGCAAGAGCAA	TGTTGGGAGCCAGATTGT
*COL10A1*	ATGCTGCCACAAATACCCTTT	GGTAGTGGGCCTTTTATGCCT
*GAPDH*	AGAAGGCTGGGGCTCATTTG	AGGGGCCATCCACAGTCTTC
*MMP3*	ATGATGAACAATGGACAAAGGA	GAGTGAAAGAGACCCAGGGA
*RUNX2*	ACCATGGTGGAGATCATCG	CGCCATGACAGTAACCACAG
*SOX9*	AACGCCGAGCTCAGCAAGA	CCGCGGCTGGTACTTGTAATC

## Data Availability

The data presented in this study are available on request from the corresponding author. The data are not publicly available because of confidentiality issues.

## References

[1] Buckwalter J.A., Mankin H.J. (1998). Articular Cartilage: Tissue Design and Chondrocyte-Matrix Interactions. Instr. Course Lect..

[2] Pearle A.D., Warren R.F., Rodeo S.A. (2005). Basic Science of Articular Cartilage and Osteoarthritis. Clin. Sports Med..

[3] World Health Organization (WHO) (2002). The World Health Report 2002–Reducing Risks, Promoting Healthy Life, Annex Table 3 Burden of disease in DALYs by Cause, Sex and Mortality Stratum in WHO Regions, Estimates for 2001, 196–197. https://www.who.int/whr/2002/en/.

[4] Yoshimura N., Muraki S., Oka H., Mabuchi A., En-Yo Y., Yoshida M., Saika A., Yoshida H., Suzuki T., Yamamoto S. (2009). Prevalence of Knee Osteoarthritis, Lumbar Spondylosis, and Osteoporosis in Japanese Men and Women: The Research on Osteoarthritis/Osteoporosis against Disability Study. J. Bone Min. Metab..

[5] Felson D.T., Naimark A., Anderson J., Kazis L., Castelli W., Meenan R.F. (1987). The Prevalence of Knee Osteoarthritis in the Elderly. The Framingham Osteoarthritis Study. Arthritis Rheum..

[6] Lawrence R.C., Felson D.T., Helmick C.G., Arnold L.M., Choi H., Deyo R.A., Gabriel S., Hirsch R., Hochberg M.C., Hunder G.G. (2008). Estimates of the Prevalence of Arthritis and Other Rheumatic Conditions in the United States: Part II. Arthritis Rheum..

[7] McAlindon T.E., Bannuru R.R., Sullivan M.C., Arden N.K., Berenbaum F., Bierma-Zeinstra S.M., Hawker G.A., Henrotin Y., Hunter D.J., Kawaguchi H. (2014). OARSI Guidelines for the Non-Surgical Management of Knee Osteoarthritis. Osteoarthr. Cartil..

[8] Zhang W., Moskowitz R.W., Nuki G., Abramson S., Altman R.D., Arden N., Bierma-Zeinstra S., Brandt K.D., Croft P., Doherty M. (2008). OARSI Recommendations for the Management of Hip and Knee Osteoarthritis, Part II: OARSI Evidence-Based, Expert Consensus Guidelines. Osteoarthr. Cartil..

[9] Harris J.D., Siston R.A., Pan X., Flanigan D.C. (2010). Autologous Chondrocyte Implantation: A Systematic Review. J. Bone Jt. Surg. Am. Vol..

[10] Nam J., Perera P., Liu J., Rath B., Deschner J., Gassner R., Butterfield T.A., Agarwal S. (2011). Sequential Alterations in Catabolic and Anabolic Gene Expression Parallel Pathological Changes during Progression of Monoiodoacetate-Induced Arthritis. PLoS ONE.

[11] Maldonado M., Nam J. (2013). The Role of Changes in Extracellular Matrix of Cartilage in the Presence of Inflammation on the Pathology of Osteoarthritis. Biomed. Res. Int..

[12] Yamato M., Okano T. (2004). Cell Sheet Engineering. Mater. Today.

[13] Nishida K., Yamato M., Hayashida Y., Watanabe K., Yamamoto K., Adachi E., Nagai S., Kikuchi A., Maeda N., Watanabe H. (2004). Corneal Reconstruction with Tissue-Engineered Cell Sheets Composed of Autologous Oral Mucosal Epithelium. N. Engl. J. Med..

[14] Ohki T., Yamato M., Murakami D., Takagi R., Yang J., Namiki H., Okano T., Takasaki K. (2006). Treatment of Oesophageal Ulcerations Using Endoscopic Transplantation of Tissue-Engineered Autologous Oral Mucosal Epithelial Cell Sheets in a Canine Model. Gut.

[15] Sawa Y., Miyagawa S., Sakaguchi T., Fujita T., Matsuyama A., Saito A., Shimizu T., Okano T. (2012). Tissue Engineered Myoblast Sheets Improved Cardiac Function Sufficiently to Discontinue LVAS in a Patient with DCM: Report of a Case. Surg. Today.

[16] Iwata T., Yamato M., Tsuchioka H., Takagi R., Mukobata S., Washio K., Okano T., Ishikawa I. (2009). Periodontal Regeneration with Multi-Layered Periodontal Ligament-Derived Cell Sheets in a Canine Model. Biomaterials.

[17] Maehara M., Sato M., Toyoda E., Takahashi T., Okada E., Kotoku T., Watanabe M. (2017). Characterization of Polydactyly-Derived Chondrocyte Sheets versus Adult Chondrocyte Sheets for Articular Cartilage Repair. Inflamm. Regen..

[18] Abe S., Nochi H., Ito H. (2016). Alloreactivity and Immunosuppressive Properties of Articular Chondrocytes from Osteoarthritic Cartilage. J. Orthop. Surg. (Hong Kong).

[19] Arthroscopic de Novo NT® Juvenile Allograft Cartilage Implantation in the Talus: A Case Presentation Elsevier Enhanced Reader. https://reader.elsevier.com/reader/sd/pii/S1067251611005928?token=622A192DC76E2E202E1C689C6650F8F36EDC0DBE5020429169FCA045999F9E590932C7478071DE98C741DD881B327855.

[20] Sato M., Yamato M., Mitani G., Takagaki T., Hamahashi K., Nakamura Y., Ishihara M., Matoba R., Kobayashi H., Okano T. (2019). Combined Surgery and Chondrocyte Cell-Sheet Transplantation Improves Clinical and Structural Outcomes in Knee Osteoarthritis. NPJ Regen. Med..

[21] Wang C.-C., Chen C.-H., Lin W.-W., Hwang S.-M., Hsieh P.C.H., Lai P.-H., Yeh Y.-C., Chang Y., Sung H.-W. (2008). Direct Intramyocardial Injection of Mesenchymal Stem Cell Sheet Fragments Improves Cardiac Functions after Infarction. Cardiovasc. Res..

[22] Ebihara G., Sato M., Yamato M., Mitani G., Kutsuna T., Nagai T., Ito S., Ukai T., Kobayashi M., Kokubo M. (2012). Cartilage Repair in Transplanted Scaffold-Free Chondrocyte Sheets Using a Minipig Model. Biomaterials.

[23] Takahashi T., Sato M., Toyoda E., Maehara M., Takizawa D., Maruki H., Tominaga A., Okada E., Okazaki K., Watanabe M. (2018). Rabbit Xenogeneic Transplantation Model for Evaluating Human Chondrocyte Sheets Used in Articular Cartilage Repair. J Tissue Eng. Regen. Med..

[24] Takatori N., Sato M., Toyoda E., Takahashi T., Okada E., Maehara M., Watanabe M. (2018). Cartilage Repair and Inhibition of the Progression of Cartilage Degeneration after Transplantation of Allogeneic Chondrocyte Sheets in a Nontraumatic Early Arthritis Model. Regen. Ther..

[25] Takizawa D., Sato M., Okada E., Takahashi T., Maehara M., Tominaga A., Sogo Y., Toyoda E., Watanabe M. (2020). Regenerative Effects of Human Chondrocyte Sheets in a Xenogeneic Transplantation Model Using Immune-deficient Rats. J. Tissue Eng. Regen. Med..

[26] Schubert T., Schlegel J., Schmid R., Opolka A., Grässel S., Humphries M., Bosserhoff A.-K. (2010). Modulation of Cartilage Differentiation by Melanoma Inhibiting Activity/Cartilage-Derived Retinoic Acid-Sensitive Protein (MIA/CD-RAP). Exp. Mol. Med..

[27] Blaney Davidson E.N., van der Kraan P.M., van den Berg W.B. (2007). TGF-β and Osteoarthritis. Osteoarthr. Cartil..

[28] Hamahashi K., Sato M., Yamato M., Kokubo M., Mitani G., Ito S., Nagai T., Ebihara G., Kutsuna T., Okano T. (2015). Studies of the Humoral Factors Produced by Layered Chondrocyte Sheets: Humoral Factors Produced by Layered Chondrocyte Sheets. J. Tissue Eng. Regen. Med..

[29] Toyoda E., Sato M., Takahashi T., Maehara M., Okada E., Wasai S., Iijima H., Nonaka K., Kawaguchi Y., Watanabe M. (2019). Transcriptomic and Proteomic Analyses Reveal the Potential Mode of Action of Chondrocyte Sheets in Hyaline Cartilage Regeneration. IJMS.

[30] Jablonski C.L., Leonard C., Salo P., Krawetz R.J. (2019). CCL2 But Not CCR2 Is Required for Spontaneous Articular Cartilage Regeneration Post-Injury. J. Orthop. Res..

[31] Goldring M.B. (2000). Osteoarthritis and Cartilage: The Role of Cytokines. Curr. Rheumatol. Rep..

[32] Rigoglou S., Papavassiliou A.G. (2013). The NF-ΚB Signalling Pathway in Osteoarthritis. Int. J. Biochem. Cell Biol..

[33] Walker P.A., Jimenez F., Gerber M.H., Aroom K.R., Shah S.K., Harting M.T., Gill B.S., Savitz S.I., Cox C.S. (2010). Effect of Needle Diameter and Flow Rate on Rat and Human Mesenchymal Stromal Cell Characterization and Viability. Tissue Eng. Part C Methods.

[34] Garvican E.R., Cree S., Bull L., Smith R.K., Dudhia J. (2014). Viability of Equine Mesenchymal Stem Cells during Transport and Implantation. Stem Cell Res. Ther..

